# The Methods of Obtaining Asepsis in Operations in Private Practice.—I

**Published:** 1898-08-13

**Authors:** W. McAdam Eccles

**Affiliations:** Assistant-Surgeon to the West London Hospital, to the City of London Truss Society, &c.


### Medical Progress and Hospital Clinics.

[*The Editor will be glad to receive offers of co-operation and contributions from members of the profession. All letters should be addressed to* The Editor, at the Office, 28 & 29, Southampton Street, Strand, London, W.C.]

### THE METHODS OF OBTAINING ASEPSIS IN OPERATIONS IN PRIVATE PRACTICE.—I.

By W. McAdam Eccles, M.S.(Lond.), F.R.C.S.(Eng.), Assistant-Surgeon to the West London Hospital, to the City of London Truss Society, &c.

It is an undoubted fact that the prick of a pin may lead to a fatal result, but it is also equally true that the largest operation wound may heal without causing much, if any, inconvenience. What is the underlying factor which produces such a difference in the two instances?

This question can be answered by all who have had any knowledge of the invasion of the tissues of the body by bacteria. It is the presence of, and the action of, these micro-organisms in operation wounds that plays so great havoc in the one case, and the absence of the same that makes the second so safe a lesion. But although this answer can be so easily given, yet the means whereby the ideal is attained of having an area free of pathogenic organisms are sometimes but little understood, and not always readily obtainable. It is well to have a very clear idea of what is indicated by the terms employed in connection with this subject. Asepsis is a word that should be strictly used, for its loose use has led to much confusion. Asepsis, as the origin of the word implies, is only to be employed when it is wished to define the state of being entirely free from the presence of micro-organisms, a condition which one must confess is not easily attained, at any rate so far as the skin of the human body is concerned. Antisepsis is the expression used when it is desired to allude to the effort made by the employment of reagents to obtain the condition of asepsis. "Aseptic surgery" is the surgery which is carried out in aseptic regions of the body, and the phrase "antiseptic surgery" is one which has hardly any meaning. The ideal is to have asepsis, and antiseptics are part of the means at the practitioner's disposal to secure this ideal.

Can asepsis in its strict sense be obtained? The answer is in the affirmative, provided that there is sufficient time allowed for its realisation. But if absolute asepsis is somewhat difficult of attainment, and involves a considerable expenditure of time, should the attempt to obtain it be given up on these accounts? By no means; for the condition of the patient cannot be made worse by employing methods to reach this end, and will, in fact, in the majority of cases be brought so near, if not completely so, to the ideal that very great good will be ensured.

It must be allowed that, although the complete removal or destruction of all the bacteria in a given area is a matter of some tediousness and difficulty, yet the methods undertaken to attempt to attain it do place the patient in a condition which is almost the best possible. Further, it is well to bear in mind that the healthy uninjured tissues of the body are perhaps the most efficient antiseptic agents that exist. Again, it ought to be an undisputed fact that no system of endeavouring to obtain asepsis can be considered to in any way approach perfection unless it can be carried out in a private dwelling equally as well as in a hospital. Much valuable time is often lost in private cases which might have been advantageously used in preparing the patient for the operation which it was known would almost certainly have to be performed.

It is the intention of this paper to indicate as clearly and briefly as possible the means which the writer considers from experience to be the best to be adopted in private cases in order to ensure asepsis. No claim will be made that the paper in any way sets forth a complete description of the subject of aseptic surgery or of the various antiseptics. First, those instances where there is time to thoroughly prepare the patient will be dealt with, and then those in which urgency is a factor. The amputation of a toe for incurable deformity will serve as a typical instance in which there is no need to perform the operation immediately. The region of the foot is, moreover, what may be termed a test area, for it is habitually extremely septic, and it is not uncommon that strict asepsis is not obtained for these small operations. It is an important fact to grasp that not only is the surface of the skin "dirty," but also that the glands in its depths are the home of countless numbers of hidden foes. It will be necessary, therefore, to cleanse the upper parts of the integument, but it is equally needful to deal with the deeper portions as well.

The following should be the line of procedure to be carried out: Supposing that the operation is to be undertaken on a Wednesday morning, on the morning of the previous Monday the patient should take a warm bath, and pay especial attention to the cleansing of the affected foot. Directly after this the nails of the foot should be cut quite short, and then the whole foot thoroughly scrubbed with soap and hot water. It is not merely sufficient to gently wash the foot, but it must be subjected to considerable friction, which may be somewhat objected to by the patient. At the end of the manipulation the surface of the skin will be found to be distinctly reddened. The washing has effected a very desirable removal of the dirt, including many of the bacteria on the surface. It is very essential that the parts in between the toes be efficiently seen to. Moreover, it is not enough to cleanse the immediate region of the operation, but it is needful to subject a wide area around it to the washing. If it be at all necessary, the parts must be shaved; in fact, this may be a desirable procedure in every case. After the scrubbing, the soap is to be washed off with some water which has been rendered sterile by boiling. Then the surface of the operation area, and for some distance around it, is to be rubbed over with either ether or turpentine; perhaps the latter is the more useful, for it is nearly always at hand and is very efficient, while ether has the disadvantage of being very inflammable. The excess of the turpentine is also to be removed by the application of boiled water. After this portion of the preparation has been successfully carried out, the whole region should be scoured with an antiseptic solution. There is no need for this to be a strong one, as a weak one will answer the purpose equally well, and be much less liable to cause irritation. Perhaps a solution of the biniodide of mercury of the strength of 1 in 1,000 is the best that can be employed. This being done, then the whole foot is to be encased in dressing consisting of double cyanide of mercury and zinc gauze moistened by being wrung out with the above solution. It is necessary that this dressing should be very securely fixed, for, if it were to shift, the skin surface already prepared would again become fouled. The dressing is left in place till the Tuesday morning, when it is removed, and the whole process as just described is repeated in every detail so far as the foot is concerned. It may be questioned as to what is the necessity for this double preparation.

It is true that while the first cleansing had the effect of rendering the surface of the skin probably nearly aseptic, yet it did not touch the depths of the integument in which lie hidden the glands with their teeming stores of bacteria. It is also a fact that these glands will continue to secrete and bring their products to the surface, together with the micro-organisms inclosed in them. The second cleansing recommended is to remove these bacteria, which have by this time arrived at the summit of the gland tubes. The dressing which has been applied after this second preparation is to be left in place until it is removed on the operation table. On the Wednesday morning, after the patient has been anæsthetised, the dressing is to be taken off the part, and the whole foot again subjected to the vigorous cleansing that it has received on the two previous occasions. From repeated trial of this method of attempting to obtain asepsis it has been found to be very efficient, and at the same time one which is not very difficult in its execution, and that even in a private house.

In a future paper the preparation of the surgeon's hands, those of his assistants, and the cleansing of the instruments will be considered, as well as the procedure to be adopted in emergency cases.

